# Coloration differences in three *Camellia reticulata* Lindl. cultivars: ‘Tongzimian’, ‘Shizitou’ and ‘Damanao’

**DOI:** 10.1186/s12870-023-04655-4

**Published:** 2024-01-02

**Authors:** Yan Qu, Zhi Ou, Qing Qing Yong, Xiang Yao, Jun Luo

**Affiliations:** Southwest Research Center for Engineering Technology of Landscape Architecture (State Forestry and Grassland Administration), Yunnan Engineering Research Center for Functional Flower Resources and Industrialization, College of Landscape Architecture and Horticulture Science, Southwest Forestry University, Kunming, Yunnan 650224 China

**Keywords:** *C. reticulata* ‘Damanao’, Mixed-color, *CHS*, *ANR*, Meristem

## Abstract

*Camellia reticulata* Lindl., also known as Yunnan Camellia, is an important ornamental plant in China, especially for its large and stunning flowers. A comprehensive understanding of their coloration mechanisms can aid breeders in developing new cultivars and improving their ornamental value; however, it is still unclear in Yunnan Camellia, especially in mixed-color flowers. In this study, we conducted metabolic and transcriptomic comparison analyses to investigate the coloration differences in three Yunnan Camellia cultivars: *C. reticulata* ‘Shizitou’ (SZT), *C. reticulata* ‘Damanao’ (MN), and *C. reticulata* ‘Tongzimian’ (TZM). Our results revealed that the initial flowering stage may play a critical role in the color change of MN. Metabolome analysis demonstrated that cyanidin was the primary anthocyanin in SZT and MN’s red region, while its content was low in TZM and MN’s white region. According to the transcriptome analysis, the anthocyanins biosynthesis pathway was reconstructed in Yunnan *Camellia*, and the low expression of *CHS* was detected in TZM and MN’s white region, while *ANR* maintained a high expression level, which may lead to the low content of cyanidin in them. Transcription factors MYBs, bHLH, and bZIP may play a key role in regulating anthocyanin-structural genes. The co-expression analysis showed that the meristem tissue may play a crucial role in the formation of the mixed white-red color in MN. Our study enriched the genetic basis of flower coloration differences in Yunnan Camellia which will be a valuable genomic resource to understanding the biology of coloration formation and for breeding the Camellia cultivars.

**Supplementary Information:**

The online version contains supplementary material available at 10.1186/s12870-023-04655-4.

## Introduction

*Camellia reticulata* Lindl., a member of the *Camellia* genus and Theaceae family, is native to China and classified as a Grade II endangered species [1]. Also known as Yunnan camellia, it is primarily distributed in Yunnan Province, southwestern Sichuan Province, and western Guizhou Province in China [2]. *C. reticulata* has over 1500 years of cultivation history in China, and many outstanding cultivars have been bred and spread around the world [1, 3]. With over 97 species in the *Camellia* genus, of which 76 are endemic to China [4]. Yunnan Province, in particular, is a significant resource source for camellia with a wide variety of over 300 species [5].

As of significant economic importance, its leaves are used for tea production, and the seeds are used for oil extraction [5]. Especially, Yunnan camellia is regarded as an exceptional ornamental plant with high economic value for its large, stunning flowers, and long blossoming season [6–8]. The flower color is a crucial ornamental trait, most Yunnan camellia blooms a red flower with few accounts of color variations. Many breeders focused on producing colorful cultivars with different colors and blossom periods [9]. *C. reticulata* ‘Tongzimian’ (TZM) is one of the few white-colored cultivars [10], and *C. reticulata* ‘Damanao’ is a bud variety of *C. reticulata* ‘Shizitou’ (SZT), while different from TZM and SZT, MN is the compound color Yunnan camellia cultivars with red and white flowers that resemble the beautiful agate patterns. A comprehensive understanding of the mechanism underlying flower color formation in Yunnan camellia can aid breeders in developing new cultivars and improving its economic value [11, 12].

Numerous studies have revealed that color formation in flowers is directly related to the dynamic of pigments [13, 14]. Anthocyanins, flavonoids, and carotenoids are crucial pigments that contribute to flower color development and protect against low-temperature stress, ultraviolet light, pests, diseases, and other injuries [15, 16]. Pigment biosynthesis pathways are found in most flowering plants, and the genetic mechanisms for coloration were identified, including the related genes for the necessary enzymes and transcriptional factors [17–19], epigenetic modifications [20, 21], and RNA interference [22]. As ornamental plants, the color formation analysis in *Camellia* species has been the subject of recent studies. Carotenoids and flavonol glycosides were identified as the primary pigments in golden yellow blooms of *C. nitidissima* [23], while cyanidin-core structure pigments produce the most dominant phenotype among wild red-flowered *Camellia* species [24]. A high level of proanthocyanidins was detected in the sepals of white tea flowers (*C. sinensis*) [25], but the anthocyanin biosynthetic pathway may be blocked in the white part of *C. japonica* ‘Tamanoura’ due to the strong suppression of chalcone synthase (*CHS*) expression [26]. Flavonoids and anthocyanins were also detected as the major determinants of flower color formation in *C. reticulata* [10], while few studies focused on the molecular coloration mechanisms of the mixed-colored presence in *C. reticulata*.

Except for MN, many mixed-color flowers are found in other species in nature, and their color formation was wildly investigated [27–29]. For instance, the regulatory mechanism of anthocyanin biosynthesis in oriental hybrid lily (pink-white color) showed that MYB transcription factors play an important role in promoting or inhibiting anthocyanin synthesis; *LvMYB5* was identified as an activator, and *LvMYB1* was identified as an inhibitor of anthocyanin synthesis [27, 28]. The pink-blue flowers of chrysanthemum could be produced by introducing a delphinidin-based anthocyanin pathway through the expression of flavonoid 3′, 5′-hydroxylase (*F3’5’H*) genes [29]. The mixed-color formation in petunia petals is mainly determined by the differential expression genes involved in the biosynthesis and modification of anthocyanins. In particular, the MYB transcription factors and the WD40 repeat protein *AN2* play a crucial role in regulating the expression of anthocyanin biosynthetic genes, while the enzymes UDP-glucose: flavonoid 3-O-glucosyltransferase (*3GT*), flavonoid 3′-hydroxylase (*F3’H*), and *F3’5’H* are involved in the modification of anthocyanin pigments [30].

The genetic mechanisms underlying color formation in Yunnan Camellia are still unclear, especially in mixed-color flowers. In this study, to analyze their color mechanisms, we conducted metabolome and transcriptome analyses on three Yunnan Camellia cultivars: MN (white-red flower), SZT (red flower), and TZM (white flower). Our study provides the metabolism and gene expression profiles during flower color formation in Yunnan Camellia and will aid in the breeding of Camellia cultivars.

## Methods and materials

### Sample collection

Petal samples were obtained from three cultivars of Yunnan Camellia, namely MN (white-red mixed flowers), SZT (red flowers), and TZM (white flowers), at different developmental stages. The plants used in this study were grown in natural tea gardens with an average annual temperature ranging from 16 to 20 °C and an average annual humidity of approximately 60%. Flower buds emerged during July–August of the first year, and the initial flowering occurred from the end of December of the first year to the beginning of January of the second year. Full bloom took place from the end of January to the beginning of February of the second year, and the last blooms were observed at the beginning of March of the second year.

For MN, samples were collected at three developmental stages (the bud, initial flowering, and full-bloom stages). Yunnan Camellia is known for its long bud stage, which typically spans about 60 days or longer. The initial flowering stage lasts for around 7 days, and the full bloom stage lasts for around 7 days. MN’s petals exhibit small white areas at the periphery during the early bud stage that gradually enlarge with development, so we collected two sets of samples during the bud stage of MN, namely bud stage 1 (early bud stage) and bud stage 3 (late bud stage). Due to the mixed color of MN’s petals at the full bloom stage, samples were separately collected from the white and red regions. SZT and TZM were used as control cultivars, and samples were only collected at the bud and full-bloom stages. To reduce the possible experimental errors, samples were collected with three replicates. Finally, 24 petal samples were collected and freeze-dried in a vacuum immediately for metabolic analysis. And 27 samples (three replicates collected from MN at bud stage 1) were collected and frozen immediately using liquid nitrogen, then stored at − 80 °C until RNA extraction.

### Identification of anthocyanins and flavonoids

The contents of anthocyanins and flavonoids were separately identified using liquid chromatography-tandem mass spectrometry (LC-MS/MS) (Sciex Triple QuadTM 6500+, Framingham, MA, USA). To prepare the analysis, petal samples were grounded into powder using a ball mill (30 Hz, 1.5 min). Data-dependent LC-MS/MS was analyzed using Analyst (v1.6.3) software.

To identify the contents of anthocyanin, approximately 50 mg of the powder sample was dissolved in 500 μL of an extraction solution containing 50% aqueous methanol and 0.1% hydrochloric acid. The solution was then vortexed for 5 min, sonicated for 5 min, and centrifuged at 12,000 r/min for 3 min. The supernatant was filtered using a microporous membrane (0.22 μm pore size) before being subjected to LC-MS/MS analysis. Reversed-phase analytical separation was carried out using 1.7 μm C18 columns. Solvent A consisted of Milli-Q® water containing 0.1% formic acid, and solvent B contained acetonitrile and 0.1% formic acid. The fractions were run on a 16 min gradient with solvent B (5% B for 6 min, 50% B for 6 min, 95% B for 2 min, and 5% B for 2 min).

For flavonoid identification, approximately 20 mg of the powder sample was mixed with 10 μL of a 4000 nmol/L internal standard mix solution and 500 μL of 70% aqueous methanol. The solution was then sonicated for 30 min and centrifuged at 4 °C for 12,000 r/min for 5 min. The supernatant was filtered with the same method as anthocyanin identification. Reversed-phase analytical separation was carried out using Waters ACQUITY UPLC HSS T3 C18 columns with 1.8 μm. Solvent A consisted of Milli-Q® water containing 0.05% formic acid, and solvent B contained acetonitrile and 0.1% formic acid. The fractions were run on a 15 min gradient with solvent B (10% B for 1 min, 20% B for 9 min, 95% B for 5 min, and 10% B for 1 min).

### RNA extraction, library preparation, and sequencing

Total RNA was extracted using Trizol reagent (Invitrogen) following the manufacturer’s protocols, and mRNA was enriched using Oligo(dT) Magnetic Beads. RNA quality was analyzed using Agilent Bioanalyzer 2100 system (Agilent Technologies, CA, USA), and RNA concentration was measured using a Qubit 2.0 Fluorometer (Life Technologies, CA, USA). RNA integrity was assessed by Agarose gel electrophoresis.

RNA library was prepared for each sample. To prepare the RNA library, RNA was fragmented by the fragmentation buffer. First-strand cDNA was synthesized using N6 random primers, and then the second strand was synthesized to obtain the cDNA. The purified cDNA was modified by end-repair, A-tail, and ligation. After fragment size selection using AMPure XP beads, PCR amplification was performed to obtain the final sequencing library. Finally, all libraries were sequenced on the Illumina HiSeq platform.

### RNA sequencing data analysis

To obtain high-quality reads, Trimmomatic (v0.36) [31] was used to remove reads with adapters and more than 20% of bases with a quality value of less than 10. Transcripts were assembled using Trinity. By mapping reads to the transcripts, only transcripts with correlation coefficients between replicate samples ≥0.8 were used for the following analysis. All qualified transcripts were annotated to function databases, including NR, SwissProt [32], KEGG (online) [33], and GO [34] databases. Only transcripts with an expression of ≥10 in at least one set of samples were used for subsequent analysis, and the average of the expression of all replicate samples was used as the gene expression. Differentially expressed transcripts were detected between two cultivars and two different developmental stages using the DEGseq R package (with fold change ≥1, FDR < 0.05, and Adjust *P*-value ≤0.001). The expression of DEGs was transformed into log2 (FPKM+ 0.01) and further standardized by Z-scorer. Hierarchical clustering analysis, principal component analysis (PCA), and k-mean clustering analysis were performed based on the normalized expression of DEGs. PCA was performed using the R package PCAtools, and K-means clustering was performed using MeV (V4.9) software. The weighted correlation network analysis (WGCNA) was performed using the R package [35]. Genes in pigment biosynthesis pathways were identified by Pathfinder Internal software based on the KEGG database.

## Results

### The initial flowering stage plays a crucial role in the color change of MN

**Fig. 1:**
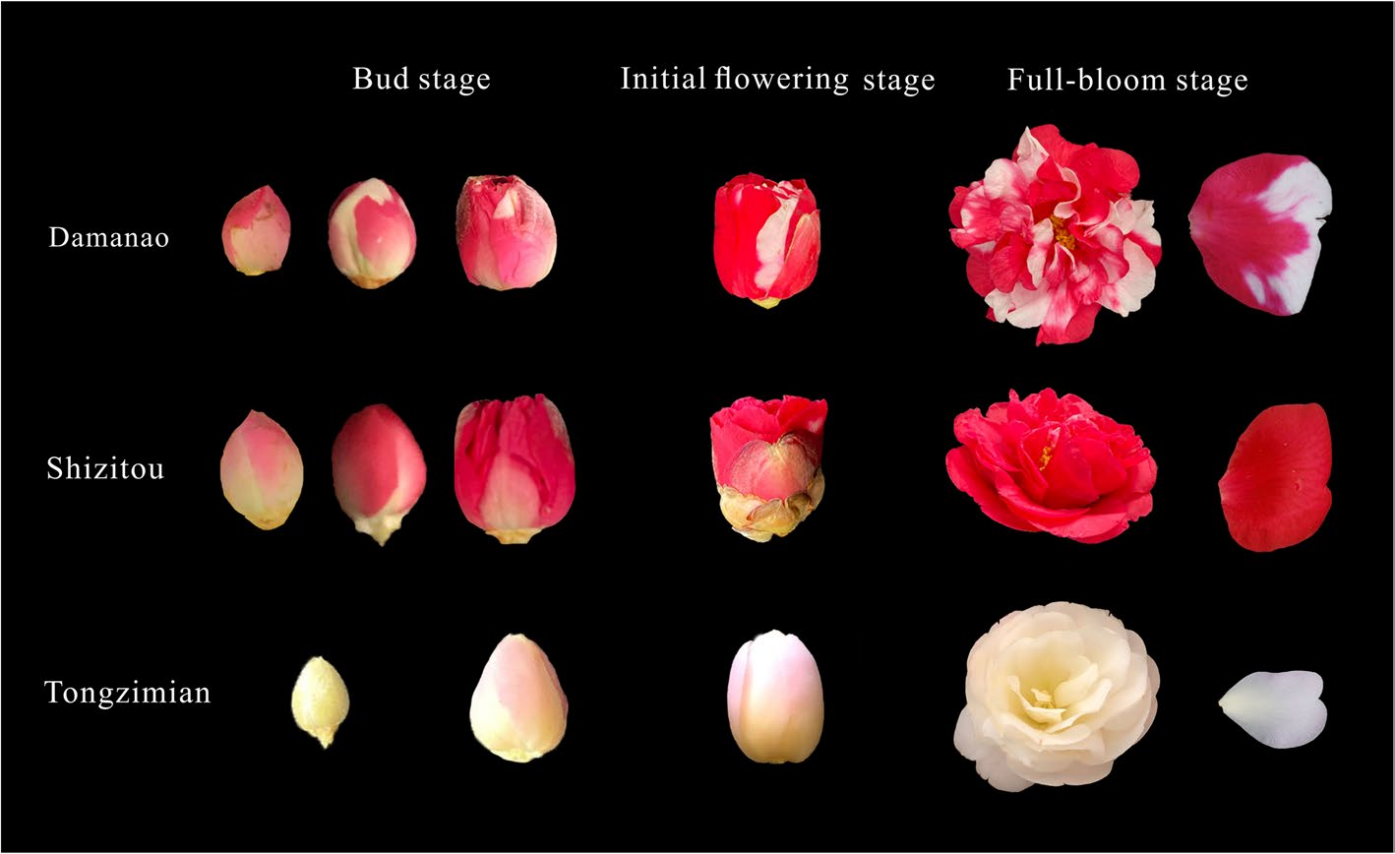
Petals at different developmental stages of TZM, SZT, and MN. Three development stages include the bud stage, initial flowering stage, and full-bloom stage. MN’s petals exhibit small white areas at the periphery during the early bud stage that gradually enlarge with development

Despite being a bud variant of SZT, MN displays a mixed coloration that differs from SZT’s pure coloration. Using the Royal colorimetric card, we observed that the color of SZT’s petals belongs to the RED GROUP throughout its development stages, with RED GROUP 53C at the bud stage, RED GROUP 46A at the initial flowering stage, and RED GROUP N45B at the full-bloom stage. In contrast, MN’s petals exhibit small white areas at the periphery during the early bud stage that gradually enlarge and stabilize during the initial flowering stage, which belongs to the WHITE GROUP 155D during its whole development. Additionally, MN also displays a red coloration throughout its development, with RED GROUP 53C at the bud stage, RED GROUP 46A at the initial flowering stage, and RED GROUP 58B at the full-bloom stage. To compare with the other Yunnan Camellia, TZM was used as the control, which is the most cultivated white variety, Although TZM appears whitish to the naked eye, the Royal colorimetric card identified its petal color as RED-PURPLE GROUP 62B at the bud stage, RED-PURPLE GROUP 62D at the first flowering stage, and RED-PURPLE GROUP 69D at full-bloom stage (Fig. 1). The red region of MN’s petals is similar to SZT’s, while the white region is different from TZM’s. The key period of color change in MN occurred during the initial flowering stage when the white region at the periphery of petals expands significantly and stabilizes. Prior to this stage, the white region is small, and its shape changes as the flower continues to develop.

### Cyanidin is the main anthocyanin in the petals of TZM, STZ, and MN

**Fig. 2:**
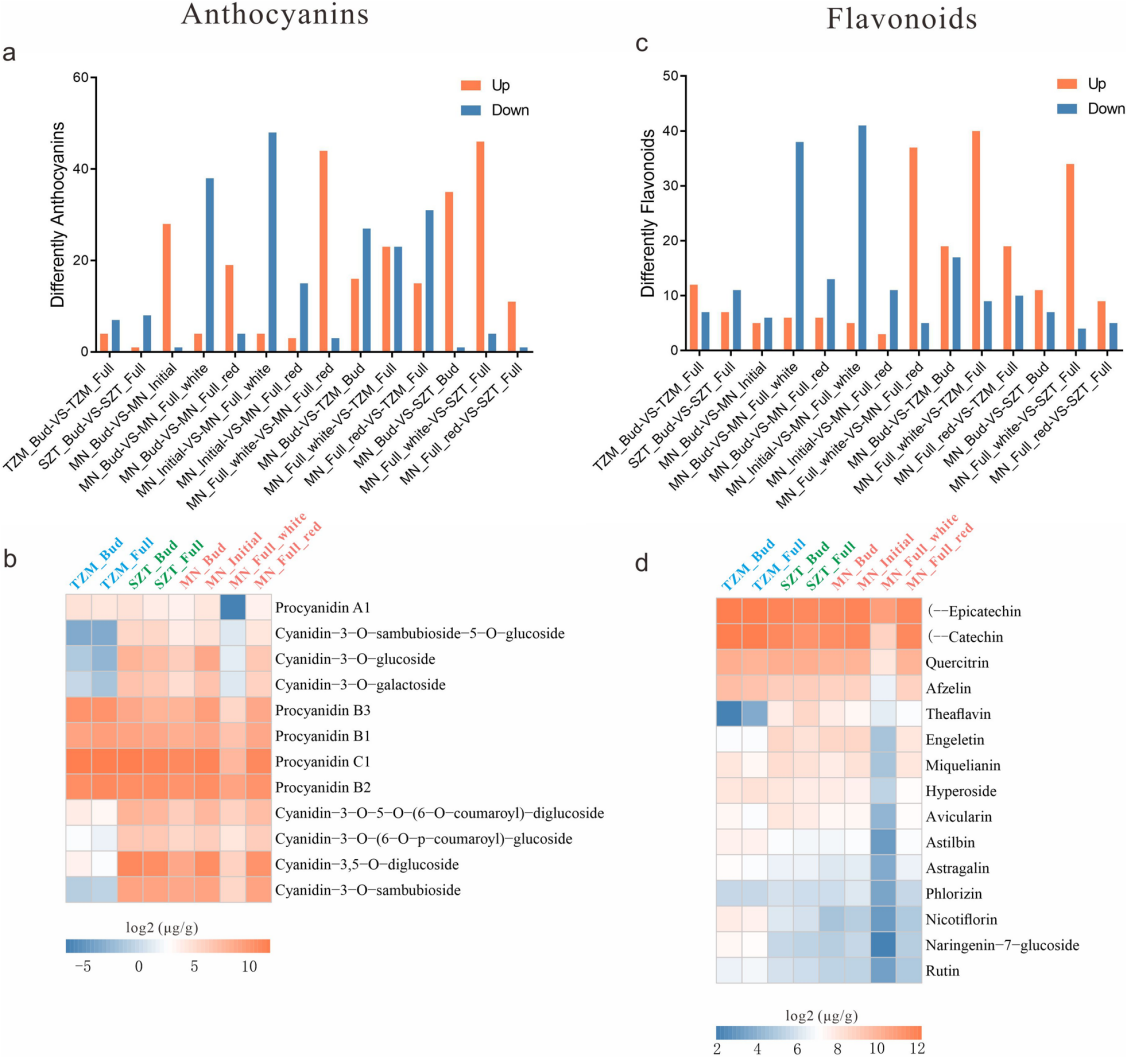
Identification of anthocyanins and flavonoids in three Yunnan Camellia cultivars. **a** Different anthocyanins in the petals of three Yunnan Camellia cultivars at different developmental stages. A total of 53 anthocyanin components were detected in all cultivars, any of which were significantly different between at least one of the two groups. The criteria for the significant difference were VIP ≥ 1, fold change ≥2, and fold change ≤0.5 for OPLS-DA. **b** Heatmap of the content and types of anthocyanins in TZM, SZT, and MN at different development stages. Only the different metabolites with high contents were displayed. **c** Different flavonoids in the petals of three Yunnan Camellia cultivars at different developmental stages. A total of 91 flavonoids were detected in the petals of the three Yunnan Camellia cultivars, 81 of which were significantly different between at least one of the two groups. **d** Heatmap of the content and types of flavonoids in TZM, SZT, and MN at different development stages

We employed LC-MS/MS to quantify anthocyanins and flavonoids in the petals of three Yunnan Camellia cultivars at different developmental stages. A total of 53 anthocyanin components were detected in all cultivars, and significant differences were observed for each of the detected anthocyanins between at least one of the two groups (Fig. 2a). The predominant anthocyanin components were procyanidin and cyanidin, which were present in glycosylated forms, with glycosylation occurring mainly at the 3- or 3,5-hydroxyl positions, and glucoside, sambubioside, and galactoside were the main types of glycosylation. Procyanidin was the anthocyanin with the highest total content in the three cultivars, with the highest content observed in TZM at the bud and full bloom stages, followed by SZT at the bud stage, MN at the initial flowering stage, and SZT at the full-bloom stage. The lowest content was observed in MN’s white region at the bloom stage. The highest total content of Cyanidin was found in SZT at the bud and full bloom stages and MN at the initial flowering stage, whereas the lowest content was found in TZM at the bud and full bloom stages. The total amount of Cyanidin in TZM at each stage was only about 1% of that in MN, while the Cyanidin content in MN’s white region at the bloom stage was just slightly higher than that in TZM, with only about 6% of total contents in all MN samples.

Among all detected cyanidins, Cyanidin-3,5-O-diglucoside and Cyanidin-3-O-sambubioside had the highest content, and the sum of the two cyanidins accounted for about 75% of the total, of which Cyanidin-3,5-O-diglucoside accounted for about 53% of the total, followed by Cyanidin-3-O-glucoside, Cyanidin-3-O-5-O-(6-O-coumaroyl)-diglucoside, Cyanidin-3-O-galactoside, Cyanidin-3-O-(6-O-p-coumaroyl)-glucoside, Cyanidin-3-O-sambubioside-5-O-glucoside, Cyanidin-3-O-galactoside, and Cyanidin-3-O-(6-O-p-coumaroyl)-glucoside. During the flower development, the contents dynamic of single cyanidin remained consistent with the total, which was the highest in SZT at both two stages as well as in MN at the initial flowering stage and the lowest in TZM at both two stages. The only slight difference was that Cyanidin-3,5-O-diglucoside, Cyanidin-3-O-5-O-(6-O coumaroyl)-diglucoside, and Cyanidin-3-O-(6-O-p-coumaroyl)-glucoside had high levels in TZM at both two stages as well as in MN at the initial flowering stage, whereas Cyanidin-3-O-sambubioside had high levels only in MN’s white region at the full-bloom stage, which was almost absent in TZM (Fig. 2b).

A total of 91 flavonoids were detected in the petals of the three Yunnan Camellia cultivars, and among them, 81 flavonoids were significantly different between at least one of the two groups (Fig. 2c). The types of detected flavonoids were mainly Flavanols (68.4%), Flavonols (23.9%), and Flavanonols (3.9%). The highest total flavonoid contents were observed in TZM at both two stages, while the lowest content was found in MN’s white region at the full-bloom stage (only about 25% of the other stages). With the exception of the bloom stage of MN, there were no significant differences in the other stages. The highest contents of Flavanols were (−)-Epicatechin and (−)-Catechin, and Quercitrin and Afzelin had the highest contents in the Flavonols. (−)- Epicatechin was the most abundant flavonoid in MN’s white region at the full-bloom stage, with the smallest differences from the other periods. Additionally, theaflavin was detected at extremely low levels in TZM at both two stages and high in all other samples (Fig. 2d).

Combining the identification of anthocyanins and flavonoids, TZM had the highest content of Procyanidin and the lowest content of Cyanidin. Cyanidin had the highest content in SZT at both stages and MN at the initial flowering stage. The total amount of Procyanidin and Cyanidin was consistent with the content of individual classifications. In MN’s white region at the bloom stage, the content of Procyanidin was the lowest, and the content of Cyanidin was slightly higher than that in TZM. The total flavonoid content was also the lowest in this stage, but the content of (−)-Epicatechin was similar to that of the other stages.

### Gene expression in the petals of TZM, STZ, and MN at different developmental stages

**Fig. 3:**
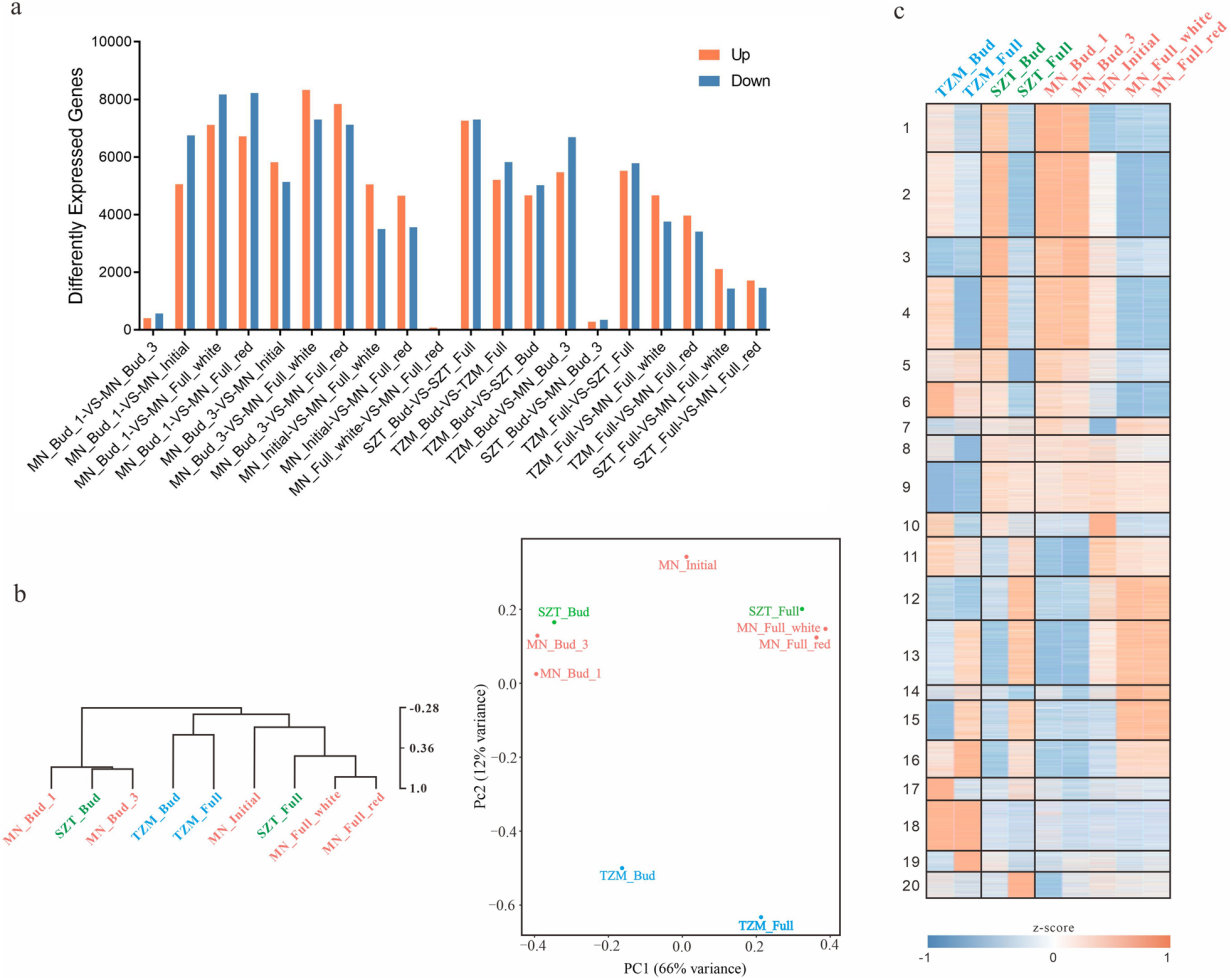
HCL, PCA, and K-means clustering for differentially expressed transcripts. **a** Statistics of differentially expressed transcripts between different samples. Differentially expressed transcripts were detected between two species and two different developmental stages with fold change ≥1, FDR < 0.05, and Adjust *P*-value ≤0.001. A total of 36,443 significantly differentially expressed transcripts were detected. **b** HCL and PCA clustering of all samples. **c** K-means clustering of all samples. The analyses of HCL, K-means, and PCA were based on significantly differentially expressed transcripts, and showed that the expression patterns of SZT and MN at the same development stage were very similar, while the initial flowering stage of MN and both two stages of TZM exhibited their unique expression patterns

To investigate the mechanism of color presentation in TZM, STZ, and MN, we collected petal samples from each cultivar at different development stages with three replicates for each time point. Total RNA was extracted from 27 samples, and RNA sequencing was performed on each sample. A total of 1205 million clean reads were obtained, with an average of 44.66 million per sample, and 225,938 transcripts were assembled, with an average length of 1160 bp, of which 65.1% were annotated in the function database (NR: 52.99%, SwissProt: 37%, KEGG: 39.82%, and GO: 44.83%). There were 225,938 transcripts successfully quantified by reads mapping, and the correlation coefficients between replicate samples within a single time-point were all ≥0.8. To minimize the effect of low expression transcripts, 55,129 transcripts with expression levels ≥5 in at least one set of samples were used in subsequent analysis. A total of 36,443 significantly differentially expressed transcripts were detected (Fig. 3a). Few differentially expressed transcripts were detected between SZT and MN at the same stage. There were also a few differentially expressed transcripts between MN bud stage 1 and bud stage 3, as well as between the white and red regions in MN at the full-bloom stage. The results of HCL, K-means, and PCA on 36,443 significantly differentially expressed transcripts showed that the expression patterns of SZT and MN at the same development stage were very similar, while the initial flowering stage of MN and both two stages of TZM exhibited their unique expression patterns.

### Chalcone synthase (*CHS*) was lowly expressed in TZM and MN’s white region at the bloom stage

**Fig. 4:**
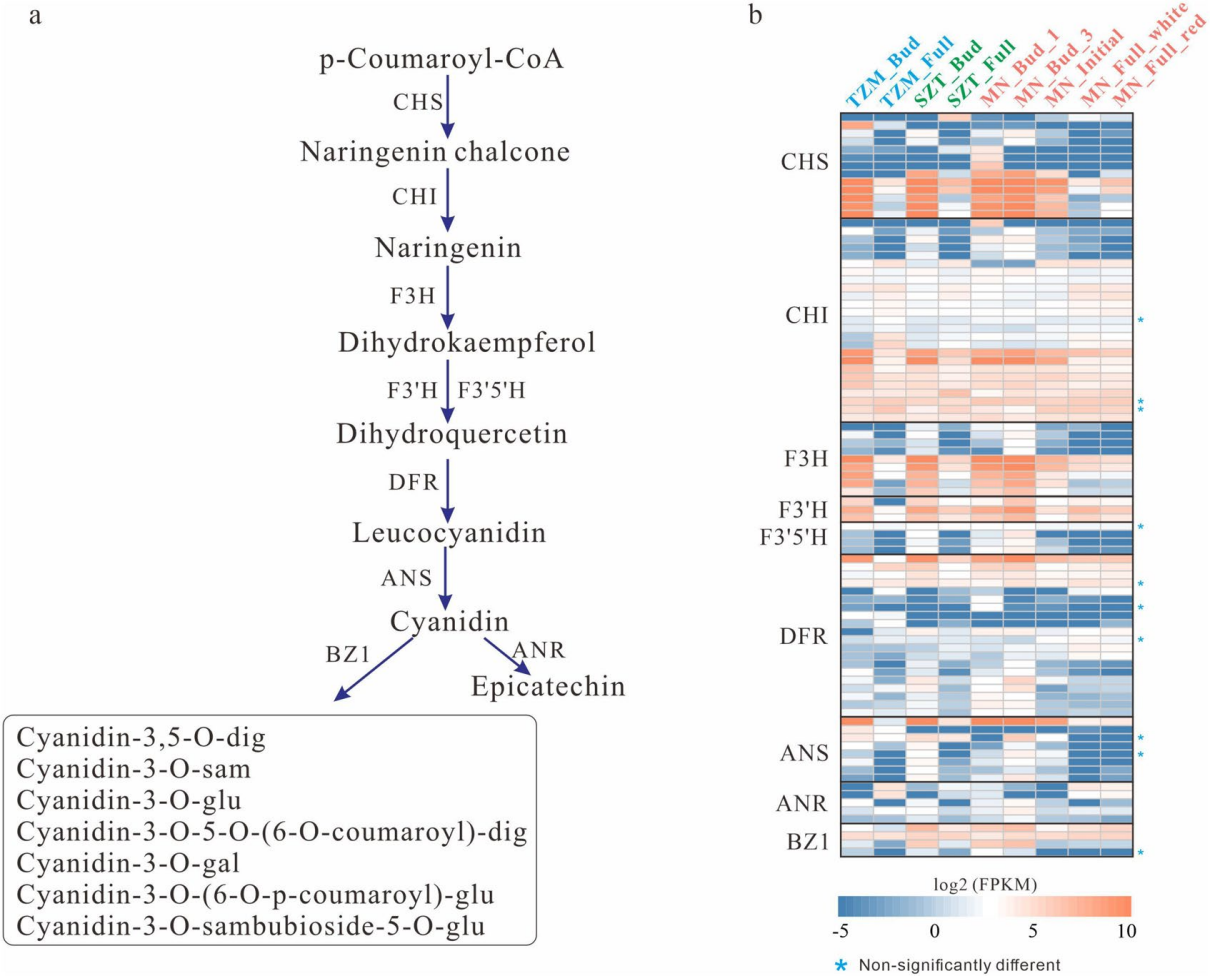
Anthocyanin synthesis pathway and the expression of involved genes. **a** Anthocyanin synthesis pathway in Yunnan Camellia. Genes were annotated using the KEGG database. **b** Heatmap of ABP-related gene expression in TZM, SZT, and MN at different development stages. Genes labeled with a blue asterisk (*) show no significant difference in comparisons of any two groups, and unlabeled genes show significant differential expression between at least two groups (padj < 0.05)

Based on the metabolome and transcriptome analysis, we reconstructed the anthocyanins biosynthesis pathway (ABP) in three Yunnan Camellia cultivars. Firstly, p-Coumaroyl-CoA was converted to dihydrokaempferol by the action of enzymes *CHS*, chalcone isomerase (*CHI*), and flavanone 3-hydroxylase (*F3H*), then to dihydroquercetin by the action of enzymes *F3’H* and *F3’5’H*, and finally to cyanidin by the action of enzymes dihydroflavonol 4-reductase (*DFR*), and anthocyanidin synthase (*ANS*). Dihydroquercetin was further converted to cyanidin by the enzymes *DFR* and *ANS*, and finally, cyanidin was either glycosylated by the enzyme Bronze-1 (*BZ1*) or converted to epicatechin by the enzyme anthocyanidin reductase (*ANR*) (Fig. 4a). Most ABP-related genes were highly expressed at the bud stage and down-regulated at the initial flowering stage, with the lowest expression at the full-bloom stage. Some genes had specific expression patterns. *CHS* was expressed at significantly lower levels in TZM and MN’s white region at the full-bloom stages than in SZT and MN’s red region at the bloom stages. *F3H* and *F3’H* were expressed at significantly lower levels in TZM than in SZT and MN at the bloom stages, while *F3’H* was expressed at lower levels in MN at the initial flowering stage than at the full-bloom stage. *ANR* had the highest expression level in TZM at the bloom stage and was also highly expressed in MN’s red and white regions at the bud and bloom stages, while its expression levels were low in SZT at both stages (Fig. 4b). In conclusion, at the bloom stage of TZM, most ABP-related genes were lowly expressed while *ANR* was highly expressed. *CHS* was expressed at low levels in MN’s white region at the bloom stage, while *ANR* was maintained at high expression levels.

### The initial flowering stage emerges as a critical period for the formation of the mixed white-red color

**Fig. 5:**
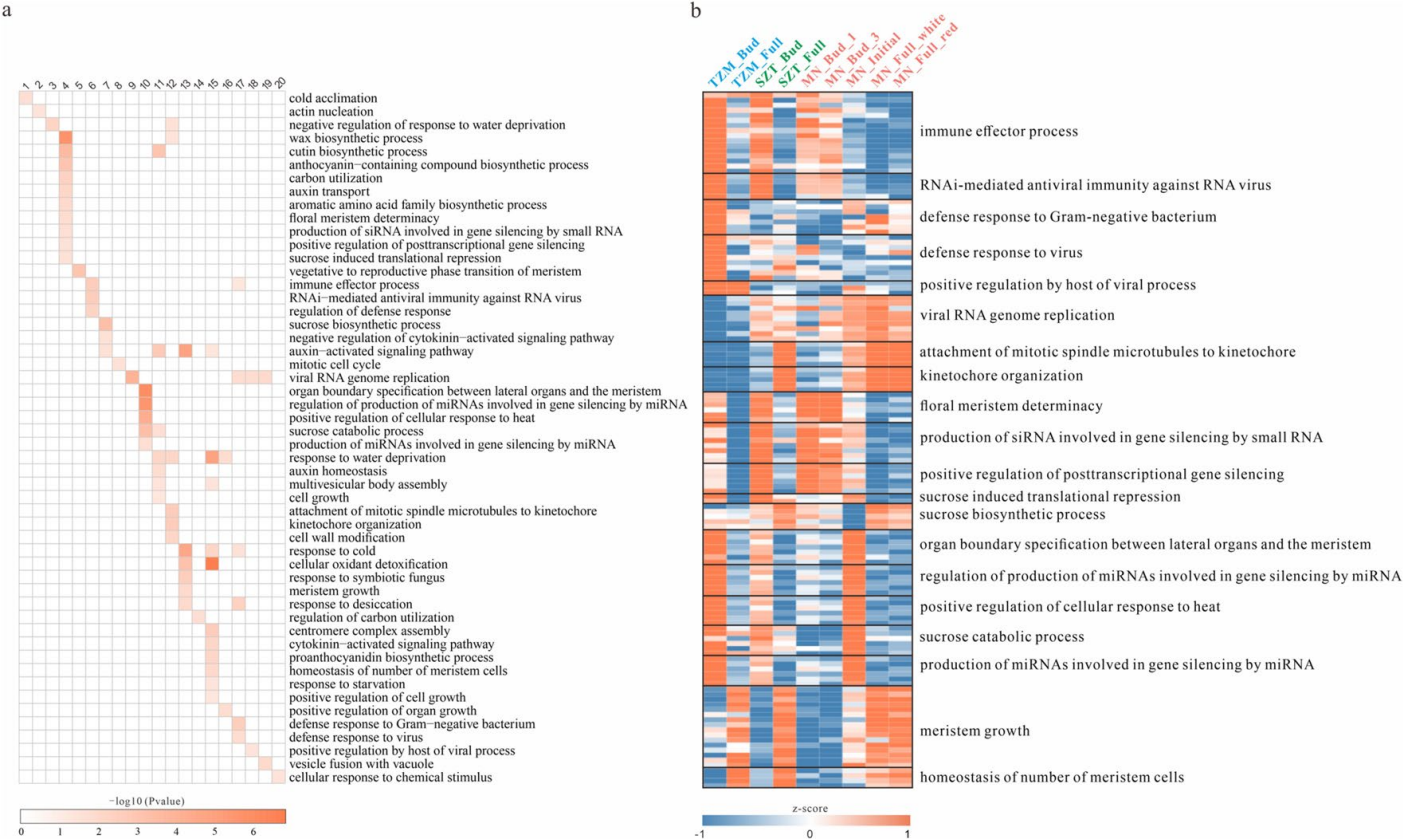
Functional enrichment of K-means clusters. **a** Functional enrichment of k-means clustering modules. **b** Heat map of specific enrichment functions corresponding to the expression of transcripts in TZM, SZT, and MN

Based on the expression patterns of the 20 k-means clusters, we found that clusters 6, 17, and 18 had higher expression levels in TZM. Functional enrichment analysis showed that these clusters were related to bacterial and viral immune responses. Cluster 6 was significantly enriched for the immune effector process and RNAi-mediated antiviral immunity against RNA virus, and clusters 17 and 18 were significantly enriched for defense response to Gram-negative bacterium, defense response to viruses, and positive regulation by host of the viral process. In contrast, clusters 3, 9, and 12 were more highly expressed in SZT and TZM. Cluster 9 was significantly enriched for viral RNA genome replication, and cluster 12 was significantly enriched for mitotic-related functions (Figs. 3c and 5a-b).

The initial flowering stage of MN was the key period for the color change, during which the boundary between the mixed red and white becomes more distinct and stable. The expression of transcripts in clusters 4, 7, and 10 was significantly different in MN between the initial flowering and full-bloom stages. The expression of transcripts in cluster 4 decreased significantly after the initial flowering stage. Transcripts in cluster 7 had the highest expression at the initial flowering stage, while transcripts in cluster 10 had the highest expression at the initial flowering stage (Fig. 3c). Cluster 4 was significantly enriched for anthocyanin synthesis, meristem-related functions, gene silencing, and sucrose-induced translational repression. Cluster 7 was significantly enriched for the sucrose biosynthetic process, while cluster 10 was significantly enriched for the heat response, gene silencing, meristem-related functions, and sucrose catabolic process (Fig. 5a, b). Furthermore, cluster 13 was significantly enriched in meristem growth, and cluster 15 was significantly enriched in the homeostasis of the number of meristem cells.

### Multiple MYB transcription factors co-expressed with *CHS* and *F3H*

**Fig. 6:**
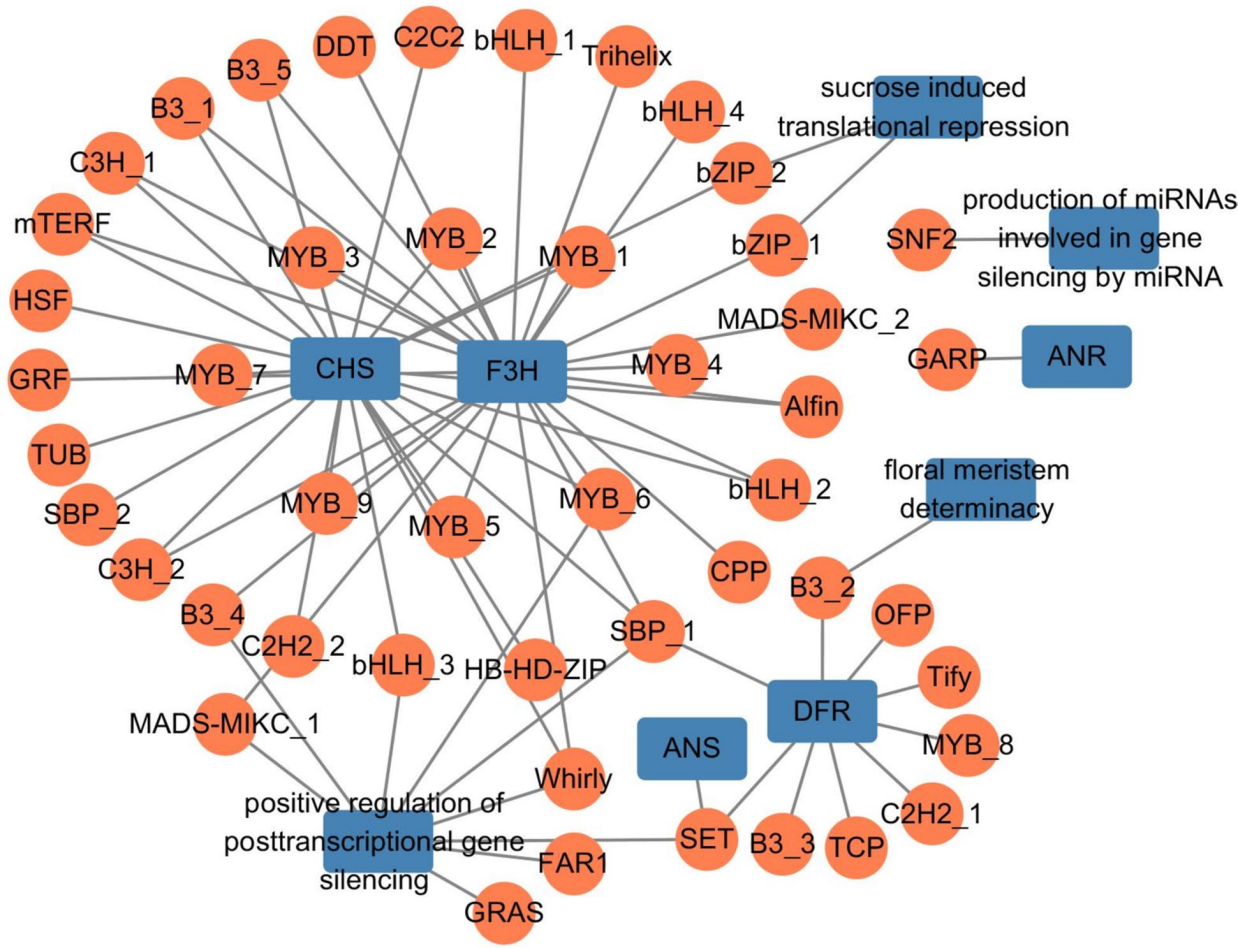
Co-expression network of ABP and specific function transcripts with transcription factors

Based on the expression patterns and functional enrichment of the 20 co-expression clusters, transcripts in clusters 4, 7, 10, 13, and 15 were strongly associated with the formation of red and white colors in MN. Cluster 4 contains several ABP-related transcripts, including *CHS*, *CHI*, *F3H*, *DFR*, and *ANS*, and cluster 15 contains transcripts corresponding to *ANR*. To investigate the co-expression relationships among the ABP-related transcripts, co-expression analysis WGCNA showed that *CHS* and *F3H* had the most co-expression relationships involving 35 transcription factors (TF) families, such as MYB, bHLH, and bZIP. Among these TF families, MYB had the most co-expression relationships, with eight TF belonging to the MYB family, six of which were co-expressed with both *CHS* and *F3H*. Positive regulation of post-transcriptional gene silencing and DFR transcripts were also abundant, and each of them co-expressed with nine TF belonging to MYB, bHLH, B3, and other families. Floral meristem determinacy transcripts were also co-expressed with B3 factors, and the B3 family was also co-expressed with *DFR*. The transcript for sucrose-induced translational repression was co-expressed with bZIP, and bZIP was co-expressed with *CHS* and *F3H* (Fig. 6, Supplymentary Table 1). Overall, co-expression results suggested that multiple MYB family TF are co-expressed with *CHS* and *F3H*, indicating they may play an important role in the regulation of anthocyanin synthesis and may relate to the formation of the mixed red and white color in MN.

## Discussions

Here, the potential coloration mechanisms of three Yunnan Camellia cultivars were first analyzed using metabolic and transcriptomic comparison methods, with a particular focus on the mixed white-red petals in MN. Petal color is economically and biologically important, while few studies have reported on Yunnan Camellia cultivars. Previous studies have identified some genes involved in anthocyanins synthesis, the pigments responsible for the red and pink colors in Camellia flowers [24, 27], but the genetic pathways that regulate their expression and interaction with other pigments are still limited, and research on white Camellia is insufficient [25, 26]. By utilizing metabolic and transcriptomic methods on three Yunnan Camellia cultivars with different colors, we were able to detect the different expression genes and metabolites that may relate to their flower coloration mechanisms.

Yunnan province provides abundant Camellia resources for the sample collection due to its diverse range of species and suitable climatic conditions. For comparative analysis of the color formation, TZM and SZT, which express white and red color flowers respectively, and MN, which possesses mixed white-red flowers, were selected as the materials (Fig. 1). All flower colors were verified using the Royal colorimetric card. To minimize the experimental errors, petals were collected from each cultivar with three replicates. Consistent results were obtained from all replicates, which strengthens the validity of our analysis.

Anthocyanins and flavonoids are usually responsible for the orange-to-blue colors that are widely distributed in many flowers, leaves, fruits, seeds, and other tissues [36, 37]. Our identification showed that it was stable of the pigment contents at the developmental stages of the same cultivars. Among the six major anthocyanins [15], cyanidin was the domain anthocyanin in three Yunnan Camellia cultivars. Typically, cyanidin is associated with pink and purple-red colors, but their colors can also change depending on the pH, co-existing colorless compounds (co-pigments), and metal ions [14]. The colorless procyanidin was markedly upregulated in white flowers [38], and our analysis also showed a high amount of procyanidin and flavonoids in TZM while extremely low contents in MN’s white region at the full bloom stages, suggesting some other factors may influence the white color presentation in MN. In addition, the white region of MN also differed significantly from other MN samples. However, the content of Epicatechin in the white region was higher than other pigments, which was similar to Epicatechin in other samples, indicating that Epicatechin may have an important role in the white presentation (Fig. 2b). In contrast, the types and contents of pigment in the red region of MN at the full-bloom stage were consistent with that in SZT, particularly cyanidin-core structure pigments were the most dominant pigments, which were also reported as the dominant pigments in other red-flowered *Camellia* species [24].

The identification of pigment contents was found to be consistent with RNA expression in ABP. By reconstructing the ABP, we observed lower expression of *CHS*, *F3H*, and *F3’H*, the key genes in ABP, in TZM and white region of MN during the full-bloom stage than in other samples (Fig. 3). The strong suppression of *CHS* expression was also reported to be the main reason for ABP blockage in the white part of *C. japonica* ‘Tamanoura’ [26]. The expression trend of ABP-related genes was highest at the bud stage and decreased gradually with flower development. However, the expression of ANR was high at the full-bloom stage in TZM and white MN that transfers cyanidin to Epicatechin, resulting in little cyanidin in the white petals during the developmental stages by combining with the low expression of *CHS*. ABP-related genes are regulated primarily at the transcriptional level by transcription factors, such as MYB, basic helix-loop-helix (bHLH), and WD-40 repeat (WDR) [39]. Our co-expression results suggested that MYBs, bHLH, and bZIP may play a key role in regulating anthocyanin-structural genes (Fig. 6), with MYB showing the most co-expression relationships with ABP-related genes, which was also consistent with previous analyses on *C. reticulata* [10].

To gain further insights into the factors influencing color formation in three cultivars, we conducted the functional enrichment of co-expression clusters. The high expression clusters in TZM were related to bacterial and viral immune responses, suggesting that TZM is more resistant to bacteria and viruses than SZT and MN. In contrast, SZT and MN had higher expression of clusters related to mitotic functions, indicating that their cells may undergo more mitotic divisions during the mid- to late developmental stages. Some clusters were significantly different in MN between the initial flowering and full-bloom stages, and the initial flowering stage of MN could be characterized by active meristem tissue, breakdown of sucrose, multiple modes of gene silencing, and heat sensitivity. Therefore, we speculate that the petals of MN sense the temperature and adjust the number of cells in the meristem accordingly during the initial flowering stage; meanwhile, gene expression is silenced and translation is inhibited in the meristem, leading to the white color of the meristem. The higher expression of ANR at the full-bloom stage further contributes to the white color formation in its petals. Moreover, cluster 13 was significantly enriched in meristem growth, and cluster 15 was significantly enriched in the homeostasis of the number of meristem cells, indicating that meristem tissue grows faster during the initial flowering stage, and the number of cells in the meristem tissue tends to be stabilized after this stage. These results also imply that the meristem tissue may play a crucial role in the formation of the mixed white-red color in MN, which could be further investigate by isolating the meristem tissue in the future.

In summary, our study showed that the initial flowering stage may be the key period for MN’s color change. Cyanidin was identified as the primary anthocyanin in SZT and MN’s red region, while it had low content in TZM and MN’s white region. According to the transcriptome analysis, ABP was reconstructed in Yunnan Camellia, and the low expression of *CHS* was detected in TZM and MN’s white region, while *ANR* maintained a high expression level, leading to the low content of cyanidin in them. Transcription factors MYBs, bHLH, and bZIP may play a key role in regulating anthocyanin-structural genes. The co-expression clusters showed functional enrichment at different cultivars, with TZM enriched in bacterial and viral immune responses, and SZT and MN exhibiting higher expression of mitotic functions. We speculate that the meristem tissue may play a crucial role in the formation of the mixed white-red color in MN, which may be influenced by temperature and would be investigated in our future studies.

### Supplementary Information

**Additional file 1: Supplementary Table 1. **Correlation coefficients and p-value of ABP and specific function transcripts with transcription factors.

## Data Availability

All data and materials mentioned in this article are available. Sequencing reads have been submitted to the NCBI Sequence Read Archive (SRA) under accession numbers SRR24413180-SRR24413206. The transcriptome assembly and expression analysis data have been submitted to the Gene Expression Omnibus (GEO) database under accession number GSE236364. The data point of contact is Yan Qu.

## References

[1] Yu JD (1985). A historical review and future development of Camellia reticulata in Yunnan. Acta Hortic Sin..

[2] Ming TL, Gu ZJ, Zhang WJ, Xie LS (2000). Monograph of the genus Camellia.

[3] Li P-R, Li Z, Zhai M (2016). Recent advances in Camellia japonica L. J Plant Genet Resour..

[4] Xia LF, Wang ZL, Feng BJ (2003). Recent studies of camellia of Kunming institute of botany.

[5] Xin T, de Riek J, Guo H, Jarvis D, Ma L, Long C (2015). Impact of traditional culture on Camellia reticulata in Yunnan, China. J Ethnobiol Ethnomed..

[6] Li JB, Hashimoto F, Shimizu K, Sakata Y (2007). Anthocyanins from red flowers of Camellia reticulata Lindl. Biosci Biotechnol Biochem..

[7] Xin T, Huang W, De Riek J, Zhang S, Ahmed S, Van Huylenbroeck J, Long C (2017). Genetic diversity, population structure, and traditional culture of Camellia reticulata. Ecol Evol..

[8] Hattan J, Shindo K, Ito T, Shibuya Y, Watanabe A, Tagaki C, Misawa N (2016). Identification of a novel hedycaryol synthase gene isolated from Camellia brevistyla flowers and floral scent of Camellia cultivars. Planta..

[9] Zhou SL, Zou X, Zhou ZQ, Liu J, Xu C, Yu J, Wang Q, Zhang DM, Wang XQ, Ge S, Sang T, Pan KY, Hong DY (2014). Multiple species of wild tree peonies gave rise to the 'king of flowers', Paeonia suffruticosa Andrews. Proc Biol Sci..

[10] Geng F, Nie R, Yang N, Cai L, Hu Y, Chen S, Cheng X, Wang Z, Chen L (2011). Integrated transcriptome and metabolome profiling of Camellia reticulata reveal mechanisms of flower color differentiation. Front Genet..

[11] Hong Y, Bai X, Sun W, Jia F (2012). The numerical classification of chrysanthemum flower color phenotype. Acta Hortic Sin..

[12] Wijayani AM, Muafi Sukwadi R (2017). Market actor’s response towards flower colours in determining the economic value of Chrysanthemum flowers. J Bus Retail Manag Res..

[13] Zhao D, Tao J (2015). Recent advances on the development and regulation of flower color in ornamental plants. Front Plant Sci..

[14] Tanaka Y, Sasaki N, Ohmiya A (2008). Biosynthesis of plant pigments: anthocyanins, betalains and carotenoids. Plant J..

[15] Mekapogu M, Vasamsetti B, Kwon OK, Ahn MS, Lim SH, Jung JA (2020). Anthocyanins in floral colors: biosynthesis and regulation in Chrysanthemum flowers. Int J Mol Sci..

[16] Chalker-Scott L (1999). Environmental significance of anthocyanins in plant stress responses. Photochem Photobiol..

[17] Holton TAC, Cornish EC (1995). Genetics and biochemistry of anthocyanin biosynthesis. Plant Cell Rep..

[18] Zheng Y, Chen Y, Liu Z, Wu H, Jiao F, Xin H, et al. Important Roles of Key Genes and Transcription Factors in Flower Color Differences of Nicotianaalata. Genes (Basel). 2021, 1976;12(12) 10.3390/genes12121976 PMC8701347 34946925

[19] Wang Y, Chen L, Yang Q, Hu Z, Guo P, Xie Q, Chen G (2022). New insight into the pigment composition and molecular mechanism of flower coloration in tulip (Tulipa gesneriana L.) cultivars with various petal colors. Plant Sci..

[20] Tang M, Xue W, Li X, Wang L, Wang M, Wang W, Yin X, Chen B, Qu X, Li J, Wu Y, Gao X, Wei X, Bu F, Zhang L, Sui Z, Ding B, Wang Y, Zhang Q, Li Y, Zhang Y (2022). Mitotically heritable epigenetic modifications of CmMYB6 control anthocyanin biosynthesis in chrysanthemum. New Phytol..

[21] Wu X, Zhou Y, Yao D, Iqbal S, Gao Z, Zhang Z (2020). DNA methylation of LDOX gene contributes to the floral colour variegation in peach. J Plant Physiol..

[22] Hao P, Liu H, Lin B, Ren Y, Huang L, Jiang L, Hua S (2022). BnaA03.ANS identified by metabolomics and RNA-seq partly played irreplaceable role in pigmentation of red rapeseed (Brassica napus). Petal Front Plant Sci..

[23] Zhou X, Li J, Zhu Y, Ni S, Chen J, Feng X (2017). De novo assembly of the Camellia nitidissima transcriptome reveals key genes of flower pigment biosynthesis. Front Plant Sci..

[24] Li JB, Hashimoto F, Shimizu K, Sakata Y (2013). Chemical taxonomy of red-flowered wild Camellia species based on floral anthocyanins. Phytochem..

[25] Joshi R, Gulati A (2011). Biochemical attributes of tea flowers (Camellia sinensis) at different developmental stages in the Kangra region of India. Sci Hortic Amst..

[26] Tateishi N, Ozaki Y, Okubo H (2010). White marginal picotee formation in the petals of Camellia japonica 'Tamanoura'. J Japan Soc Horticult Sci..

[27] Yang J, Meng J, Liu X, Hu J, Zhu Y, Zhao Y, Jia G, He H, Yuan T (2021). Integrated mRNA and small RNA sequencing reveals a regulatory network associated with flower color in oriental hybrid lily. Plant Physiol Biochem..

[28] Yin X, Zhang Y, Zhang L, Wang B, Zhao Y, Irfan M, Chen L, Feng Y (2021). Regulation of MYB Transcription Factors of Anthocyanin Synthesis in Lily Flowers. Front Plant Sci..

[29] Brugliera F, Tao GQ, Tems U, Kalc G, Mouradova E, Price K, Stevenson K, Nakamura N, Stacey I, Katsumoto Y, Tanaka Y, Mason JG (2013). Violet/blue chrysanthemums--metabolic engineering of the anthocyanin biosynthetic pathway results in novel petal colors. Plant Cell Physiol..

[30] Morita Y, Hoshino A (2018). Recent advances in flower color variation and patterning of Japanese morning glory and petunia. Breed Sci..

[31] Bolger AM, Lohse M, Usadel B (2014). Trimmomatic: a flexible trimmer for Illumina sequence data. Bioinform..

[32] Bairoch A, Apweiler R. The SWISS-PROT protein sequence database and its supplement TrEMBL in 2000. Nucleic Acids Res. 2000;28(1) 10.1093/nar/28.1.45 PMC102476 10592178

[33] Kanehisa M, Goto S. KEGG: Kyoto encyclopedia of genes and genomes. Nucleic Acids Res. 2000;28(1) 10.1093/nar/28.1.27 PMC102409 10592173

[34] Harris MA, CJ Ireland A, et al. The gene ontology (GO) database and informatics resource. Nucleic Acids Res. 2004;32(suppl 1) 10.1093/nar/gkh036 PMC308770 14681407

[35] Langfelder P, Horvath S (2008). WGCNA: an R package for weighted correlation network analysis. BMC Bioinform..

[36] Dudek B, Warskulat AC, Schneider B (2016). The occurrence of flavonoids and related compounds in flower sections of Papaver nudicaule. Plants (Basel)..

[37] Alappat B (2020). Anthocyanin pigments: beyond aesthetics. Molecules..

[38] Lang X, Li N, Li L, Zhang S. Integrated metabolome and transcriptome analysis uncovers the role of anthocyanin metabolism in Michelia maudiae. Int J Genomics. 2019;2019:4393905. 10.1155/2019/4393905. eCollection 2019. 10.1155/2019/4393905 PMC6874964 31781588

[39] Patzlaff A, McInnis S, Courtenay A, Surman C, Newman LJ, Smith C, Bevan MW, Mansfield S, Whetten RW, Sederoff RR (2003). Characterisation of a pine MYB that regulates lignification. Plant J..

